# Research trends of nanomaterials in *Helicobacter pylori*: a bibliometric analysis from 2003 to 2023

**DOI:** 10.3389/fphar.2025.1546395

**Published:** 2025-03-28

**Authors:** Yeqing Lei, Tao Chen, Qin Du, Weihua Yu

**Affiliations:** ^1^ Department of Gastroenterology, The Fourth Affiliated Hospital of School of Medicine, and International School of Medicine, International Institutes of Medicine, Zhejiang University, Yiwu, China; ^2^ Department of Gastroenterology, The Second Affiliated Hospital, Zhejiang University School of Medicine, Hangzhou, Zhejiang, China

**Keywords:** bibliometric analysis, *Helicobacter pylori*, nanomaterial, research trend, treatment

## Abstract

**Background:**

*H. pylori* infects approximately half of the global population and is associated with numerous diseases, posing a significant public health challenge. Recently, there has been increasing focus on researching nanomaterials for *H. pylori*. This study aims to visually evaluate the current status and trends of nanomaterials in *H. pylori* research by bibliometric analysis.

**Methods:**

*H. pylori*-related nanomaterials publications were retrieved from WoSCC and articles meeting the criteria were included in the analysis. The data was analyzed by Microsoft Excel, CiteSpace, and VOS viewer.

**Results:**

This bibliometric analysis included 177 publications on *H. pylori* and nanomaterials from 2003 to 2023. The study revealed a consistent increase in publications and citations. China leads in the number of publications, citation frequency, and maintains close relations with other countries. *The International Journal of Biological Macromolecules* and *Biomaterials* are the leading journals. Yu-hsin Lin is the most contributory scholar. Recent years have seen the special nanoparticles and targeted drug delivery remain a burgeoning research area.

**Conclusion:**

We conducted a bibliometric analysis of *H. pylori*-related nanomaterials research and identified the current research direction and frontier in the application of nanomaterials for *H. pylori*.

## 1 Introduction

*Helicobacter pylori* (*H. pylori*) is a microaerophilic, gram-negative, flagellated, curved rod-shaped bacterium with the unique ability to colonize the human gastric mucosa, infecting half of the global population. (Malfertheiner et al., 2022). *H. pylori* infection is linked to multiple gastrointestinal diseases, including chronic gastritis, peptic ulcers, and notably serving as a predominant risk factor for gastric cancer. (Robinson and Atherton, 2021; Wang et al., 2014). Additionally, numerous extra-gastric diseases are also closely related to *H. pylori*, posing a considerable public health challenge. (Gravina et al., 2020). *H. pylori* can spread through various routes, including fecal-oral, oral-oral, and gastric-oral transmission means, as well as between humans and from animals to humans. (Duan et al., 2023). A recent meta-analysis revealed notable regional differences in *H. pylori* infection rates, ranging from 18.9% in Switzerland to 87.7% in Nigeria, attributed to geographical, economic, and social factors. (Hooi et al., 2017). Furthermore, a large-scale study in China reported an average individual *H. pylori* infection rate of 40.66% among families, with an average family infection rate of 71.21%. (Zhou et al., 2023). Due to its highly infectious nature and complex pathogenicity, *H. pylori* was classified as a Class I carcinogen for gastric cancer, the fifth most common tumor and the fourth leading cause of cancer-related death globally. (P and ylori, 1994; Sung et al., 2021). Therefore, the current Maastricht VI/Florence consensus report recommends that all patients with *H. pylori* should receive treatment, regardless of clinical symptoms. (Malfertheiner et al., 2022). Since its discovery by Barry Marshall and Robin Warren in 1983, a combination of antibiotics and antacids has been developed for the effective treatment and eradication of *H. pylori* infection.

Both the mainstream empirical treatment with bismuth quadruple therapy and the currently popular high-dose dual therapy have proven effective in treating *H. pylori* infection in adult population. (Malfertheiner et al., 2022; Zhu et al., 2020). Additionally, vonoprazan, a novel potassium-competitive acid blocker (p-CAB), exerts a stronger and more sustained inhibitory effect on intragastric acid secretion compared with classical proton pump inhibitors (PPIs), providing more treatment options. (Qian et al., 2023; Murakami et al., 2016). However, the escalating incidence of antibiotic resistance poses a significant obstacle for *H. pylori* management. (Tshibangu-Kabamba and Yamaoka, 2021). In recent decades, there has been a significant increase in antibiotic resistance of *H. pylori* to key antibiotics like clarithromycin, metronidazole, and levofloxacin in all WHO regions. (Savoldi et al., 2018). A recent survey indicated that the primary resistance to clarithromycin and metronidazole in the Asia-Pacific region was 30% and 61%, respectively. (Hong et al., 2024). Therefore, novel and effective approaches are requisite for *H. pylori* eradication.

Nanomaterials, typically defined as materials with diameters from 1 to 100 nm, have been extensively investigated for diagnosing and treating gastric cancer. (Jeevanandam et al., 2018; Li R. et al., 2017). Nanomaterials also show great potential in treating *H. pylori*, a major cause of gastric cancer. (Wang et al., 2014; Safarov et al., 2019). Previous studies have revealed that nanomaterials can be coupled with *H. pylori* binding molecules to facilitate targeted therapy in various ways and show great potential for *H. pylori* eradication. (Lopes et al., 2014; Chitas et al., 2024).

Bibliometric analysis is a modern scientific methodology that employs mathematical and statistical techniques based on public literature databases to evaluate the output of countries, institutions, authors, and journals within a specific research domain, thereby enabling the examination of research focus directions and trends in that particular field. (Chen et al., 2012). Previous bibliometric studies have covered topics like *H. pylori* and drug resistance, microbiota, and immunotherapy. (Shi et al., 2022; Li M. et al., 2023; Li Y. et al., 2023). This bibliometric analysis includes 177 studies focusing on *H. pylori*-related nanomaterials from 2003 to 2023, presenting the current state and future trends of this field.

## 2 Methods

### 2.1 Data collection

The data were obtained from the Web of Science Core Collection (WoSCC), an online database providing standardized and up-to-date reference data for scientific research. The search strategy combined the topics of *H. pylori* and nanomaterial using the following search formula: (TS = (“*H. pylori*” OR “*Campylobacter pylori*” OR “*H. pylori*” OR “*Campylobacter pylori subsp. Pylori*” OR “*Campylobacter pyloridis*” OR “*Campylobacter pylori*”)) AND TS = (nano*). The search term “nano*” was employed to capture words that start with “nano,” such as nanoparticle, nanomaterial, nanocarrier, nanocomposite, and nanotechnology, among others. Initially, publications were limited to English-language original articles and review articles, excluding other types of literature like conference proceedings and letters. In the second step, the titles and abstracts of the remaining studies were carefully assessed to ensure they specifically focused on the application of nanomaterials in *H. pylori*. Two authors (Yeqing Lei and Tao Chen) independently evaluated the articles, and any disagreements were resolved with the third author (Qin Du). As of 1 October 2024, a total of 177 English-language articles on *H. pylori* and nanomaterials were retrieved from 2003 to 2023, including both original articles and review articles. The study selection process is presented in Supplementary Figure S1.

### 2.2 Data analysis

The records of the retrieved papers, including title, authors, country, institutions, journal, keywords, and references, were exported into a plain text file and subsequently imported into CiteSpace (version.6.3. R1 Advanced), VOS viewer (version.1.6.20), and Microsoft Excel 2016 (Microsoft, Washington, USA) for qualitative and quantitative analysis. Microsoft Excel 2016 was used for database management and annual publication analysis. CiteSpace, a Java-based tool developed by Professor Chen from Drexel University, was used for reference collaboration, dual maps of journals, literature bursts, and keyword bursts analysis. (Chen and Song, 2019). VOS viewer, created by Nees Jan van Eck et al., is designed for co-citation and co-occurrence analysis and generates visual representations. (van Eck and Waltman, 2010).

## 3 Results

### 3.1 Annual publication and citation analysis

**FIGURE 1:**
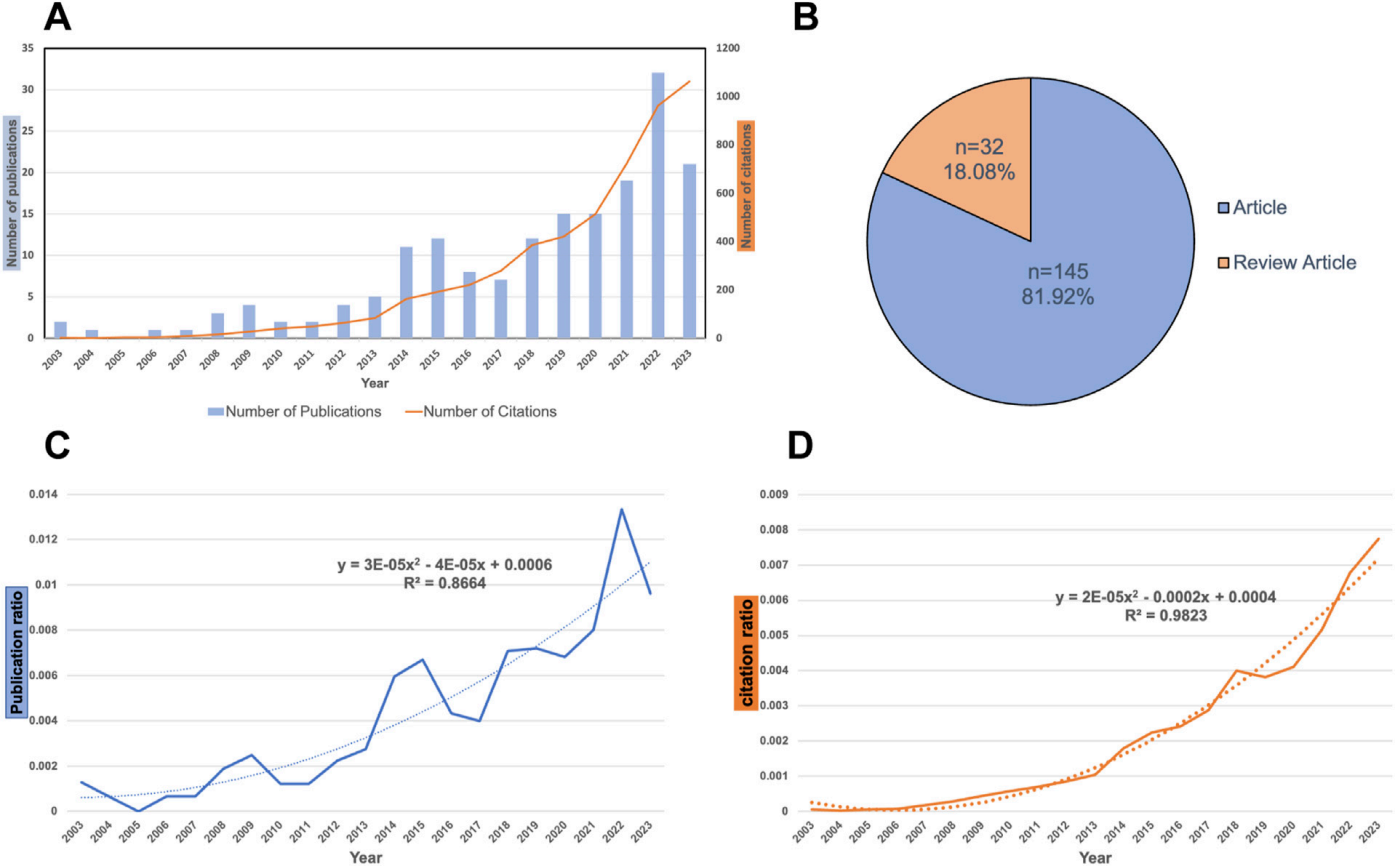
Publication and citation analysis on *H. pylori*-related nanomaterials research. **(A)** Trend in the publications related to *H. pylori* and nanomaterial research from 2003 to 2023. **(B)** Document types composition. 177 studies were analyzed. Of those, 145 (81.92%) were original research articles, and 32 (18.08%) were reviews. **(C)** Trends in the publication rate of *H. pylori-*related nanomaterials from 2003 to 2023. We use the following search formula: [TS = (“*Helicobacter pylori*” OR “*Campylobacter pylori*” OR “*H. pylori*” OR “*Campylobacter pylori subsp. Pylori*” OR “*Campylobacter pyloridis*” OR “*Campylobacter pylori*”)] to search the English-language literature on general *H. pylori* from 2003 to 2023 in the WoSCC database, including articles and review articles. Next, we documented the number of publications and citations for general *H. pylori* research each year and calculated the ratios of publications and citations for *H. pylori*-related nanomaterial research compared to general *H. pylori* research. The curve depicts the ratio of the number of *H. pylori*-related nanomaterial studies to general *H. pylori* studies from 2003 to 2023. The dashed line indicates the tendency in the proportion of the number of studies related to the *H. pylori* nanomaterial to the number of general *H. pylori* studies. **(D)** Trend in the citation ratio of *H. pylori-*related nanomaterials from 2003 to 2023. The curve shows the change in the ratio of citations from *H. pylori*-related nanomaterial studies to those from general *H. pylori* studies between 2003 and 2023. The dashed line shows the tendency in the proportion of *H. pylori* nanomaterial related citations in general *H. pylori* research.

Figure 1A presents the annual trends in publication output and citation frequency. The number of publications and citation trends offers insights into the advancements and trends in research on *H. pylori* and nanomaterials. From 2003 to 2023, the number of publications and citations on *H. pylori*-related nanomaterial research was relatively low but exhibited an overall upward trend. It is worth noting that 2022 has the highest publication number (32), while 2023 has the highest citation number (1062). On average, 8.4 papers were published annually, each receiving 29.4 citations. Of these, 145 (81.92%) were original research articles, and 32 (18.08%) were review articles, as shown in Figure 1B.

To effectively highlight advancements in *H. pylori*-related nanomaterial research, we initially searched the WoSCC for English-language literature on general *H. pylori* studies from 2003 to 2023, including original articles and review articles, using the following search formula: (TS = (“*H. pylori*” OR “*Campylobacter pylori*” OR “*H. pylori*” OR “*Campylobacter pylori subsp. Pylori*” OR “*Campylobacter pyloridis*” OR “*Campylobacter pylori*”)). Next, we documented the annual number of publications and citations for general *H. pylori* research and calculated the ratios of publications and citations for *H. pylori*-related nanomaterial research compared to general *H. pylori* research. The results are presented in Figures 1C, D. Figure 1C shows an increasing publication ratio trend (*R* ^2^ = 0.8664), with fluctuations in the ratio between *H. pylori*-related nanomaterial research and general *H. pylori* research. Overall, the number of *H. pylori*-related nanomaterial studies increased from 2003 to 2023. Figure 1D demonstrates a strong fit (*R* ^2^ = 0.9823) and a clear annual increase in the citation proportion of *H. pylori*-related nanomaterial research. In conclusion, the growth in publications and citations for *H. pylori*-related nanomaterial studies exceeds that of general *H. pylori* studies.

### 3.2 Country and institution contribution analysis

A total of 177 papers were contributed by researchers from 37 countries. Supplementary Table S1 lists the top 10 countries by publications, citations, and total link strength. Link strength indicates the frequency of international collaboration, with higher scores reflecting broader cooperation. Notably, China and India rank first and second in both publications and citations. Additionally, China, Egypt, and Saudi Arabia are among the top three in total link strength. For publications, China leads with 82, followed by India with 26 and Egypt with 20. For citations, China leads with 2840, followed by India (1225) and the United States (687). For total link strength ranking, China ranks first with 28, followed by Egypt (21) and Saudi Arabia (18).

**FIGURE 2:**
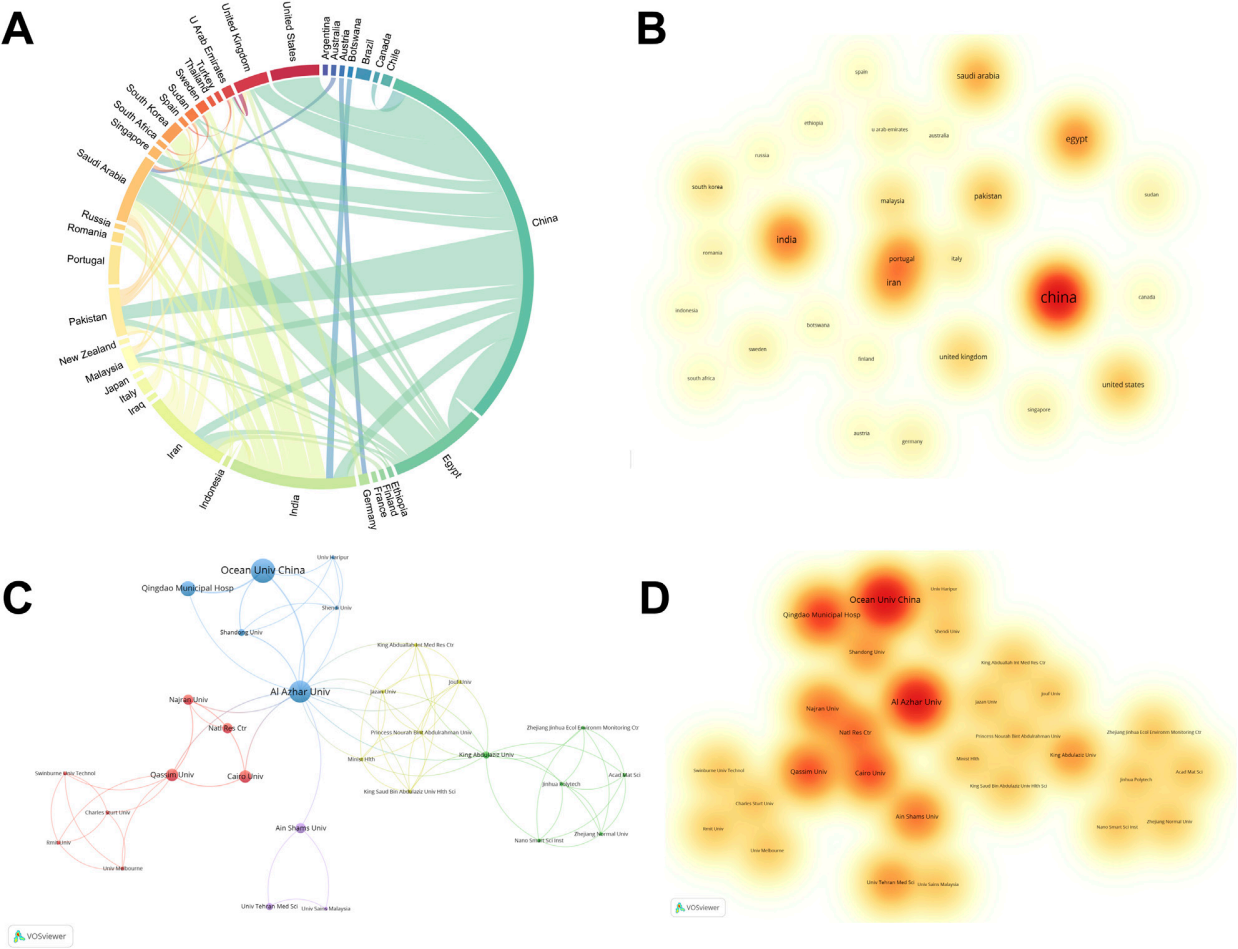
Country and institution analysis. **(A)** Country co-occurrence map of *H. pylori*-related nanomaterial research. The arc length directly correlates with the volume of publications from each country, and the thickness of the connection reflects the strength of the partnership. **(B)** The density visualization map of countries in *H. pylori*-related nanomaterial research. The magnitude of the word, the magnitude of the round, and the opacity of red are positively correlated with the number of publications. **(C)** Institutions cluster analysis of *H. pylori*-related nanomaterial research. Each node symbolizes an institution, and the size of the circle is in proportion to the quantity of articles published by that institution. The connections among nodes signify the degree of correlation, and a greater number of connections indicates more cooperation. **(D)** The density visualization map of institutions in *H. pylori*-related nanomaterial research. The magnitude of the word, the magnitude of the round, and the opacity of red are positively correlated with the number of 200 publications.

The chord diagram in Figure 2A illustrates the collaborative relationship among various countries. Each arc’s length represents the number of publications from each country, and the thickness of the connecting chords indicates the intensity of their collaborations. China leads in publications, followed by India and Egypt. Additionally, China also has the strongest ties with Egypt and the most extensive collaborations with other countries. Figure 2B shows a visual density map of countries, highlighting those with high publication counts. China and India dominate with the highest publication counts, making prominent contributions in this field.

293 institutions from various countries participated in 177 studies. Supplementary Table S2 shows the top 10 institutions ranked by publications and citation frequency. Four of the top 10 institutions are from China, three from Egypt, and three from Saudi Arabia. The Ocean University of China leads with 9 publications, closely followed by Al-Azhar University and China Medical University, each with 8 publications. Chinese institutions have played a crucial role in advancing research on *H. pylori*-related nanomaterials. The top 10 institutions published 43 articles, representing 24.3% of the total. In citation frequency, the Ocean University of China ranked first with 240 citations, followed by Qingdao Municipal Hospital (166) and Al-Azhar University (159). Additionally, we performed a cluster analysis to illustrate the collaboration patterns among these institutions. Figure 2C shows a network map of major institutions, divided into five clusters by color. The largest blue cluster includes Al-Azhar University, the Ocean University of China, and Qingdao Municipal Hospital. The red cluster includes Cairo University, Qassim University, and Najran University. The green cluster mainly comprises King Abdulaziz University. The yellow cluster includes Azan University, King Abduallah International Medical Research Center, and Jouf University. The purple cluster includes Ain Shams University, the Universiti Sains Malaysia, and the Tehran University of Medical Sciences. Figure 2D presents a visual density map of major institutions, highlighting those with high publication counts. Overall, Al-Azhar University and the Ocean University of China stand out with the highest publication counts.

### 3.3 Author and co-cited author contribution analysis

**FIGURE 3:**
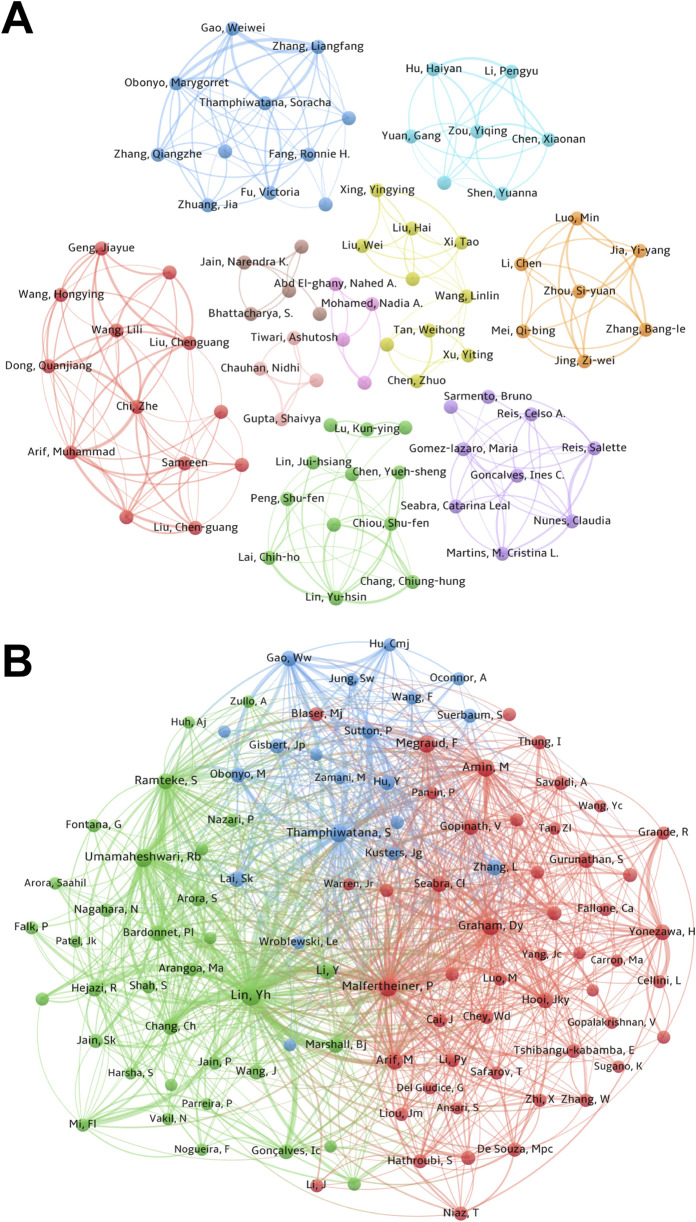
The author and co-cited author contribution analysis. **(A)** The co-author map in the field of *H. pylori*-related nanomaterial. Each circle symbolizes a unique author, and the connecting lines between the circles signify the interrelationships among the authors. The nodes of the same color represent the same cluster. **(B)** The co-cited authors map in the field of *H. pylori*-related nanomaterial. Nodes of the same color belong to the same cluster. Nodes of distinct colors denote the authors of various collaborative relationships. The size of the word, circle, and connection thickness is directly proportional to the frequency of co-citation.

More than 900 researchers participated in 177 studies. Supplementary Table S3 lists the top 10 most productive authors and their countries. Yu-hsin Lin takes the lead with 8 publications, followed by Muhammad Arif (7) and Zhe Chi (7). Notably, four of the top 10 authors are from China, and three are from the United States. Figure 3A shows the co-author cluster analysis, indicating scattered collaborations with limited close connections. Yu-hsin Lin and Muhammad Arif are active nodes in the green and red networks, respectively, demonstrating their extensive collaborations with various authors.

A statistical and cluster analysis of co-cited authors in *H. pylori*-related nanomaterial studies is shown in Supplementary Table S3 and Figure 3B. Among the top 10 co-cited authors, Yu-hsin Lin stands out with 84 co-citations and a total link strength of 1672. Notably, three of the top 10 co-cited authors are from China, highlighting the country’s significant contribution. Peter Malfertheiner follows with 52 co-citations and 1040 total link strength, and RB Umamaheshwari with 48 co-citations and 878 total link strength. Yu-hsin Lin ranks first in publications, co-citations, and total link strength, highlighting significant contributions and widespread recognition in this field. Figure 3B shows the cluster analysis of co-cited authors, illustrating the network of academic partnerships and collaborations. Eight of the top 10 co-cited authors are active in the red and green clusters, indicating that the research direction is mainly divided into two orientations with close connections.

### 3.4 Journal and co-cited journal contribution analysis

**FIGURE 4:**
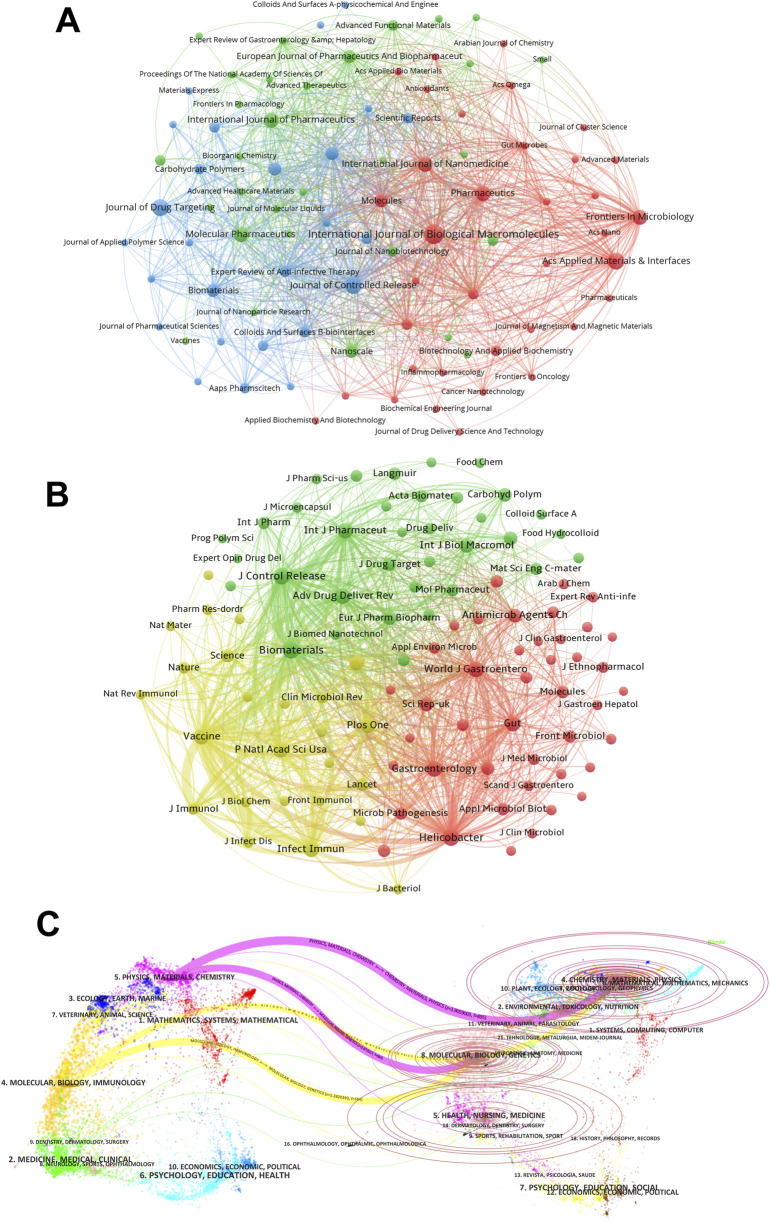
The journal and co-cited journal contribution analysis. **(A)** The journal map in the field of *H. pylori*-related nanomaterial. Each node symbolizes a journal. Nodes of the same color indicate that they belong to the same cluster. The size of the node represents the number of publications, and the link denotes the citation relationship between journals. The thickness of the link reflects the citation intensity between journals. **(B)** The co-cited-journal map in the field of *H. pylori*-related nanomaterial. Nodes of the identical color pertain to the same cluster. The size of the node denotes the co-citation frequency of the journal, and the link represents the co-citation relationship among journals. The thickness of the link mirrors the co-citation intensity between journals. **(C)** The dual-map overlay of journals on *H. pylori*-related nanomaterial. The labels situated on the left side of the dual map stand for citing journals, while those on the right represent cited journals. The colored paths denote citation relationships.

A total of 117 journals have published articles on *H. pylori*-related nanomaterial research. Supplementary Table S4 ranks the top 10 journals by publication and citation counts. These journals are all indexed in Q1 or Q2 of the Journal Citation Reports (JCR), with six having an impact factor (IF) over 5. In terms of publication volume, the top three most productive journals are the International Journal of Biological Macromolecules (7), Acs Applied Materials & Interfaces (5), and the Journal of Controlled Release (5). Together, they contributed 17 publications, accounting for 9.6% of the total. We then utilize cluster analysis to categorize the journals into three groups, as shown in Figure 4A. Nodes of the same color belong to the same cluster. Node size reflects the number of publications, and links represent citation relationships. Link thickness indicates the strength of these relationships. We observe that there are numerous intimate and extensive interconnections within the journal network. Among the top 10 journals based on article count, five are in the red cluster, including the International Journal of Biological Macromolecules and Acs Applied Materials & Interfaces. The blue cluster includes the Journal of Controlled Release and the Journal of Drug Targeting. The green cluster contains the International Journal of Pharmaceutics and Molecular Pharmaceutics.

Supplementary Table S4 lists the top 10 co-cited journals, all ranked in Q1 or Q2 of JCR. Five journals have an IF exceeding 10. *Biomaterials* leads with IF = 12.8 and 225 citations, followed by *the Journal of Controlled Release* (IF = 10.5, 219 citations) and *Helicobacter* (IF = 4.3, 205 citations). *The Journal of Controlled Release* is the only one in the top 5 for both publications and citations. Figure 4B shows the co-citation relationships through cluster analysis and visualization. Eight of the top 10 co-cited journals are prominent in the red and blue clusters, indicating a robust and interconnected citation network.

The dual-map overlay of journals (Figure 4C) visually displays the distribution of topics and the evolution of citation trajectories. Labels on the left represent citing journals and those on the right represent cited journals. Colored paths depict the citation relationships. Figure 4C highlights two primary citation paths for *H. pylori*-related nanomaterials research. The physics/materials/chemistry (purple line) and the molecular/biology/immunology (yellow line) journals, known as research frontiers, are considerably influenced by the molecular/biology/genetics and the chemistry/materials/physics journals, known as the knowledge base. the strongest citation link is from the physics/materials/chemistry to the chemistry/materials/physics journals.

### 3.5 Keyword analysis

**FIGURE 5:**
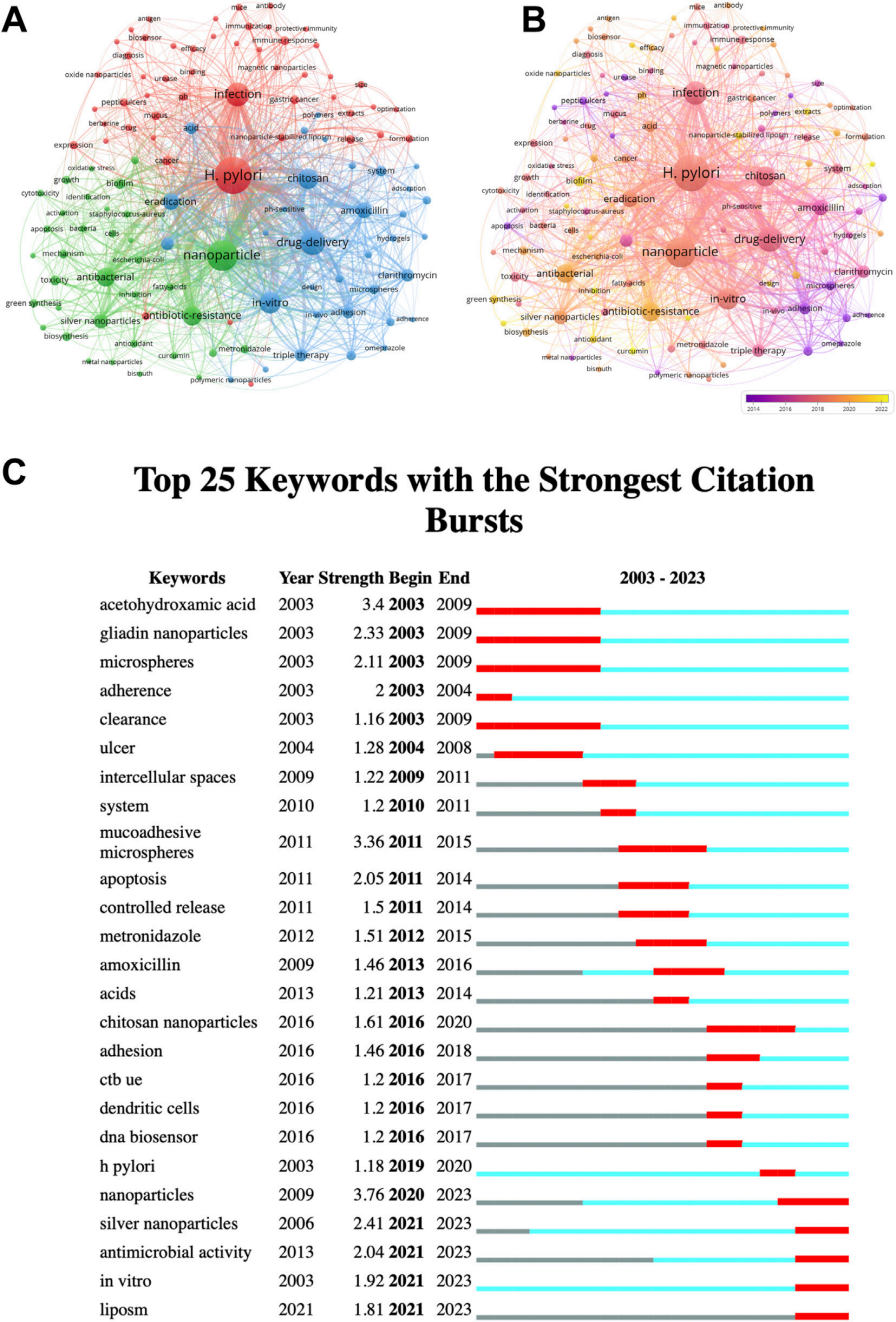
The keyword analysis. **(A)** The network visualization map of keyword analysis. Keywords are categorized into three groups. Each keyword is depicted as a node. The nodes of distinct colors stand for different clusters, and the size of the nodes indicates their frequency. The lines connecting the nodes signify the co-occurrence relationship. **(B)** The overlay visualization map of keyword analysis. Different colors represent different mean published years. Purple indicates an earlier time while yellow indicates a more recent one. **(C)** Top 25 keywords with the strongest citation bursts. These keywords are sorted by starting year. The blue bars signify the publication of the reference, while the red bars indicate citation burst.

The keywords offer a concise summary of the article’s core content. Supplementary Table S5 lists the top 20 keywords for *H. pylori*-related nanomaterial studies. One keyword appears over 100 times, ten appear more than 20 times. Two keywords have a total link strength over 500, while fifteen exceed 100. *H. pylori* (129 occurrences) and nanoparticle (91 occurrences) rank first and second. We then perform cluster analysis and create visualization maps using VOS viewer. As illustrated in Figure 5A, the cluster network comprises three closely interconnected clusters. The red cluster focuses on *H. pylori* infection and related diseases, such as gastric cancer and peptic ulcers. The blue cluster centers on drug delivery methods and targeted drugs like amoxicillin and clarithromycin. The green cluster focuses on the antibacterial effects of nanoparticles amid widespread antibiotic resistance. Figure 5B shows the prevalent keywords for each mean published year using color-coded clusters. Purple indicates earlier years, while yellow represents recent years. Since 2020, keywords like “antibiotic resistance,” “bio-synthesis,” “biofilm,” “green synthesis,” and “special nanoparticles” have gained significant attention. This suggests that, in the context of widespread antibiotic resistance, the role of biofilms and the synthesis of special nanomaterials like silver nanoparticles, nanoparticle-stabilized liposomes, and oxide nanoparticles, may become key areas for future *H. pylori* research.

*Subsequently*, we used CiteSpace to analyze the burst detection of keywords. The top 25 keywords with the strongest citation bursts are listed in Figure 5C. The earliest keywords are “active chronic gastritis” and “*campylobacter pylori*.” Recently, there has been increased interest in “silver nanoparticles,” “antimicrobial activity,” “liposome,” and “*in vitro*.” These keywords highlight the current research focuses and advancements in nanomaterials related to *H. pylori*. Therefore, we predict that special nanoparticles, particularly silver nanoparticles, and drug delivery methods will be pivotal in future *H. pylori*-related nanoparticle research.

### 3.6 Reference analysis

Supplementary Table S6 presents the top 10 most highly cited references on *H. pylori*-related nanomaterial studies. One article has received over 200 citations, six have more than 100 citations, and the remaining three have more than 90 citations. Notably, 8 out of 10 articles were published in or after 2010. The most cited paper, with 235 citations, is “Green Synthesis of Silver Nanoparticles through Reduction with Solanum xanthocarpum L. Berry Extract: Characterization, Antimicrobial and Urease Inhibitory Activities against *H. pylori*” by Muhammad Amin et al., published in the International Journal of Molecular Sciences in 2012. (Amin et al., 2012). This article reveals that silver nanoparticles synthesized from Solanum xanthocarpum berry extract have anti-*H. pylori* and urease activities, suggesting potential applications in treating *H. pylori* infections. The most recently published article is “*In vivo* activation of pH-responsive oxidase-like graphitic nanozymes for selective killing of *H. pylori*” by Lufeng Zhang et al., published in *Nature Communications* in 2021, with 97 citations. (Zhang et al., 2021). In this study, the authors developed a pH-responsive graphitic nanozyme that can be activated *in vivo* for targeted treatment of *H. pylori*. In a mouse model, the nanozyme shows high antibacterial activity against *H. pylori* with minimal side effects on normal tissues and symbiotic bacteria.

To better present the citations of the top 10 references, we have plotted their annual citations since publication in Supplementary Figure S2. The size of the circles indicates the number of citations for each paper, with larger circle representing higher citation rate and greater influence. Notably, five articles published since 2015 have garnered considerable citations, including studies by Saravanan et al. (2018), Zhang et al. (2021). Conversely, the citations for the other five articles published before 2015 have declined in recent years.

**FIGURE 6:**
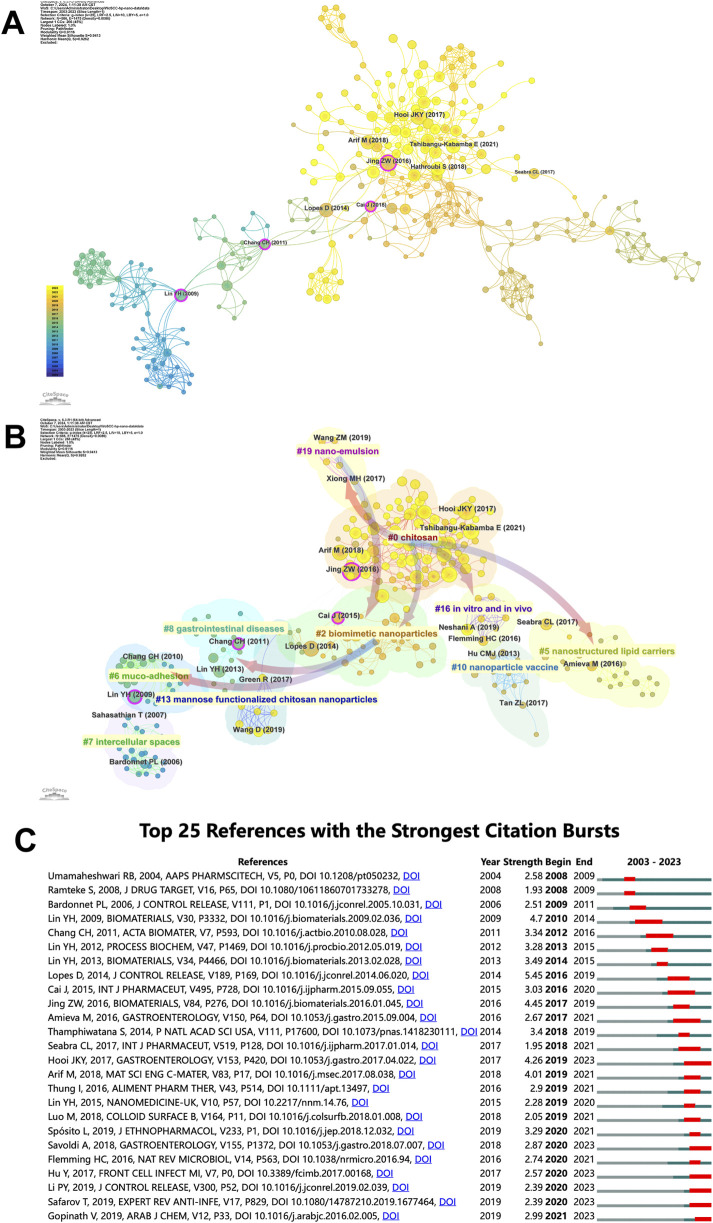
Analysis of *H. pylori*-related nanomaterial references. **(A)** Co-cited references network related to *H. pylori*-related nanomaterial. The identical color indicates that the references are attributed to the same cluster, and the link represents the co-occurrence relationship among the references. The size of the node is proportional to the frequency of the article being co-cited. **(B)** Cluster view of references in *H. pylori*-related nanomaterial. Clustering of references based on similarity between references, including #0 Chitosan, #2 Biomimetic nanoparticles, #5 Nanostructured lipid carriers, #6 Muco-adhesion, #7 Intercellular spaces, #8 Gastrointestinal diseases, #10 Nanoparticle vaccine, #13 Mannose functionalized chitosan nanoparticles, #16 *In vitro* and *in vivo*, and #19 Nano-emulsion. **(C)** Top 25 references with the strongest citation bursts. The green bars signify the publication of the reference, while the red bars indicate citation burst.

Figure 6A shows the main frequently co-cited references. Figure 6B illustrates the relationships between these references, grouped into clusters based on their associations. Each cluster is color-coded to represent its unique degree of association. The largest cluster (#0) features the highest number of publications, with “chitosan” being the most frequently mentioned keyword. The primary citations within this cluster include work by Jing et al. (2016), Hooi et al. (2017). In terms of timeline, the early research areas in *H. pylori* and nanomaterial include clusters #6 (Muco-adhesion) and #8 (Gastrointestinal diseases), which subsequently evolved into #2 (Biomimetic nanoparticles). #2 and the subsequent #5 (Nanostructured lipid carriers), #16 (*In vitro* and *in vivo*), and #19 (Nano-emulsion) further evolved into #0. The current research frontier focuses on #0 (Chitosan) and #5 (Nanostructured lipid carriers).

Figure 6C presents the top 25 most influential references based on their citation bursts. The first citation burst occurred in 2008, with subsequent bursts occurring annually from 2016 to 2021. Notably, the paper titled “Eradication of *H. pylori*: Past, present and future,” by Daniela Lopes et al., published in *the Journal of Controlled Release* in 2014, had the strongest citation burst (Strength = 5.45), lasting until 2019. (Lopes et al., 2014). Another article with a significant burst (Strength = 4.7) is “Development of pH-responsive chitosan/heparin nanoparticles for stomach-specific anti-*H. pylori* therapy,” authored by Yu-Hsin Lin et al. in Biomaterials in 2009. (Lin et al., 2009). Between 2019 and 2021, twelve of the 25 most influential citation bursts occurred, with six focusing on nanoparticles targeting *H. pylori* treatment and continuing until 2023. This suggests that research on *H. pylori* nanoparticles remains an active and ongoing area of study.

## 4 Discussion

### 4.1 General information

This study explores the evolution of *H. pylori* and nanomaterials literature from 2003 to 2023 through a comprehensive bibliometric analysis, using tools like VOS viewer and CiteSpace to visually present emerging trends and cutting-edge research priorities in the application of nanomaterials in *H. pylori*.

Our analysis reveals an increasing trend in the publication and citations of *H. pylori*-related nanomaterials research over the past few decades. Compared to general *H. pylori* research, the progress in *H. pylori*-related nanomaterials research outpaces that of general *H. pylori* research. China stands out as the leading contributor in this field, considering metrics like publication count, citation frequency, and national collaboration. According to Supplementary Table S2, four of the top 10 institutions by publication count and the top two by citation frequency are located in China, highlighting China’s significant investment in this area. The Ocean University of China and Yu-hsin Lin is the most influential institution and author, respectively. The International Journal of Biological Macromolecules has published the most articles on this topic, while Biomaterials is the most cited journal. The paper titled “Green Synthesis of Silver Nanoparticles through Reduction with Solanum xanthocarpum L. Berry Extract: Characterization, Antimicrobial and Urease Inhibitory Activities against *H. pylori*” published in the International Journal of Molecular Sciences in 2012 by Muhammad Amin et al. is the most frequently cited and has laid the groundwork for many subsequent studies. The paper titled “*In vivo* activation of pH-responsive oxidase-like graphitic nanozymes for selective killing of *H. pylori*” by Lufeng Zhang et al., published in *Nature Communications* in 2021, is the most recently published article among the top 10 most highly cited references, representing the current research hotspot.

### 4.2 Exploring research hotspots through keyword analysis

We explored the evolving trends in this research area through keyword analysis. The keywords “*H. pylori*” and “nanoparticle,” as components of the search formulation, formed the basis, center, and key of the research. Notably, keywords related to “eradication,” “drug delivery,” “antibiotic resistance,” and “chitosan” play an essential role in connecting to other clusters. This indicates that while researchers have diverse research orientations and focus, the fundamental topic and goal remain consistent. High-frequency terms like “drug delivery,” “eradication” and “antibiotic resistance” suggest that nano-related drug delivery systems might be a future research direction for *H. pylori* treatment. Earlier studies emphasized the mucosal adhesion of nanoparticles, while recent studies focused more on “green synthesis” and “biofilm”. Furthermore, recent keyword burst analysis revealed a significant increase in terms like “silver nanoparticles,” “antimicrobial activity,” “liposome,” and “*in vitro*” over the past 3 years, offering an insight into research trends. Finally, keyword analysis suggests that nano-related drug delivery systems, the role of biofilms, and the synthesis of special nanomaterials are key research priorities in studies related to *H. pylori* and nanomaterials.

### 4.3 Exploring research hotspots through reference analysis

Co-cited references are those cited by researchers in common. The aim of co-cited reference analysis is to identify the shared research foundation between *H. pylori* and nanomaterials. In this study, highly co-cited references are classified into ten clusters with different colors by CiteSpace. Timeline analysis shows that early research clusters #6 (Muco-adhesion) and #8 (Gastrointestinal diseases) evolved into #2 (Biomimetic nanoparticles). #2 and #5 (Nanostructured lipid carriers), #16 (*In vitro* and *in vivo*), and #19 (Nano-emulsion) jointly evolve into #0 (Chitosan). The current research focus area is on the closely related #0 (Chitosan) and #5 (Nanostructured lipid carriers), indicating potential future research directions in *H. pylori* and nanomaterials.

The purpose of analyzing highly cited references is to determine research focus areas and trends in *H. pylori* and nanomaterials. We find that six of the top 10 highly cited references are associated with the green synthesis of nanomaterials with anti-*H. pylori* activity. Three articles focus on targeted drug delivery of nanomaterials, and one article addresses the anti-*H. pylori* adhesion activity of nanomaterials. These findings imply that the development of *H. pylori*-related nanomaterials falls into three main directions: direct inhibition of *H. pylori* growth, targeted drug delivery, and inhibition of *H. pylori* adhesion to gastric epithelial cells. These areas represent future research hotspots, especially the development of nanomaterials with anti-*H. pylori* activity, which may replace traditional antibacterial drugs.

Reference burst analysis also provides information on the hotspots and trends in the field. From Figure 6C,six of the top 25 most influential citation bursts continuing until 2023. These bursts focus on *H. pylori* prevalence, antibiotic resistance, and novel eradication therapies like the application of nanomaterials. Among them, Pengyu Li et al. developed a novel nanoparticle that can improve the eradication rate of *H. pylori* biofilm. (Li P. et al., 2019). Furthermore, Gopinath, V et al. developed a gold nanoparticle that can inhibit multi-drug resistant *H. pylori* strains and show excellent biocompatibility. (Gopinath et al., 2019). Similar to keyword analysis, reference analysis confirms that nanomaterial-related drug delivery systems and the synthesis of specific nanomaterials are future research directions.

### 4.4 Research status and future perspective on *H. pylori-*related nanomaterials

#### 4.4.1 Detection: non-invasive and *in situ*

Given the high prevalence of *H. pylori* infection, rapid, painless, and accurate detection is essential. Current mainstream diagnostic methods are either invasive or prone to interference from medications (Malfertheiner et al., 2023). Special nanomaterials have great potential to enable more accurate *in situ* detection of *H. pylori*. Several researchers have conducted interesting investigations in this field. Yunjie Li et al. have designed stable magnetic graphitic nano-capsules (MGNs) specifically for *in situ* targeted MRI detection of *H. pylori*. (Li Y. et al., 2017). *H. pylori*, which is specifically recognized and bound by MGNs, was directly detected through T2-weighted MR Imaging and Raman imaging of gastric tissue. Additionally, the concept of nano-(bio)sensors, leveraging the advantages of nanotechnology, has been developed and held significant potential. The biosensor integrates a biorecognition component with a transducer to convert biological activity into a measurable signal. (de la Escosura-Muñiz et al., 2010). Utkarsh Jain et al. developed an ultra-sensitive immune sensor using three nanomaterials to achieve a wide range of CagA antigen detection, enabling ultra-sensitive and early detection of *H. pylori* in non-invasively collected clinical samples (Jain et al., 2020). Furthermore, nano-sensing technology can also detect other *H. pylori* antigens like VacA, BabA, HopQ, and urease (Hubalek et al., 2007; Gupta et al., 2020; Jaradat et al., 2023; Saxena et al., 2022). The outcomes of these efforts demonstrate that nanomaterials hold significant potential for the reliable detection of *H. pylori*, offering an efficient, simplified, and more dependable alternative that expands the target population for testing.

#### 4.4.2 Drug delivery: precision delivery and targeted release

The harsh stomach conditions can degrade most antibiotics, thereby reducing their delivery efficiency, preventing the achievement of effective antimicrobial concentrations locally and ultimately leading to eradication failure (Lai et al., 2022). Improving drug delivery efficiency may be a promising approach to address the challenges associated with anti-*H. pylori* therapy. Lipid-based nanoparticles (LNPs) are widely utilized in antimicrobial therapy as drug delivery systems (Pinilla et al., 2021). They possess a composition akin to cell membranes, exhibiting high biocompatibility and minimal toxicity (Naseri et al., 2015). Furthermore, when integrated with other materials, LNPs demonstrate superior features including targeting capabilities and controlled drug release, leading to enhanced drug delivery efficiency and reduced drug resistance incidence in *H. pylori* infections (Li M. et al., 2019). Muhammad Irfan Alam et al. created a targeted liposomal drug delivery system using furazolidone and N-acetylcysteine, achieving complete *H. pylori* eradication in just 2.5 h (Alam et al., 2024). Similarly,Shu-Jyuan Yang et al. designed a complex nanoparticle combining chitosan, poly (acrylic acid), superparamagnetic iron oxide nanoparticles and amoxicillin, which can avoid therapeutic failures due to easy degradation of antibiotics and side effects resulting from high-dose administration, and enable rapid and continuous release of amoxicillin between the mucus layer and the gastric epithelium for direct action against *H. pylori* (Yang et al., 2020). In summary, developing appropriate drug delivery systems for targeted and effective antimicrobial delivery offers a promising solution with lower risks and higher benefits compared to creating new antimicrobials.

#### 4.4.3 Antimicrobial mechanisms: direct elimination and indirect disruption

Biofilm formation is a significant contributor to the inefficacy of treatment methods. Inhibition or disruption of biofilms is crucial for eradicating *H. pylori* infection. Regarding the inhibition of *H. pylori* biofilm formation, silver nanoparticles stand out as the most representative agent (Almeida Furquim de Camargo et al., 2020; Gopalakrishnan et al., 2020). The rubropunctatin-silver composite nanoliposomes developed by Zhao et al. (2024) demonstrated the capability to effectively eliminate mature *H. pylori* biofilms and inhibit biofilm regeneration in laboratory settings. These nanoliposomes showed enhanced efficacy in eradicating *H. pylori* and protecting the mucosa, surpassing traditional triple therapy. Gopalakrishnan et al. (2020) found that N-acyl homoserine lactonase-stabilized silver nanoparticles disrupt quorum sensing by degrading signaling molecules, reducing biofilm formation and improving treatment outcomes. Additionally, Xiao et al. (2022) used AlpB, a crucial outer membrane protein in *H. pylori* biofilm formation, as a recognition element for screening anti-biofilm drugs and developed an innovative biosensor incorporating AlpB, colloidal gold, nanoporous gold, Nafion-reduced graphene oxide, and a glassy carbon electrode. This biosensor accurately identifies anti-biofilm drugs and evaluates their sensitivity and binding affinity of various agents to AlpB, providing valuable insights into drug efficacy.

Furthermore, gold nanoparticles (AuNPs) are commonly used for eradicating *H. pylori* by adhering to its surface and disrupting the membrane (Zhi et al., 2019; Thamphiwatana et al., 2013). Developed pH-sensitive acid-sensitive cis-aconitic anhydride-modified anti-*H. pylori*-conjugated gold nanostars (GNS@Ab) that actively target and effectively eliminate *H. pylori in vivo* in model animals under near-infrared laser irradiation (Zhi et al., 2019). Additionally, AuNPs can penetrate cells and generate reactive oxygen species (ROS), disrupting the metabolic processes of *H. pylori* and leading to its eradication (Thamphiwatana et al., 2013; Zhang et al., 2020). The excessive ROS generated by nanomaterials can specifically induce oxidative stress, causing damage to biomolecules such as proteins (leading to oxidative carbonylation), lipids (resulting in peroxidation), and nucleic acids (DNA/RNA, causing strand breaks),ultimately leading to cell necrosis, apoptosis, or mutagenesis (Yu et al., 2020). Due to the unique anti*-H. pylori* mechanism, nanomaterial may also be suitable for developing antibiotic free anti-*H. pylori* agents.

### 4.5 Main challenges in the application of nanomaterials for *H. pylori*

Although nanomaterials hold great potential, they face several challenges in clinical application like gastrointestinal degradation, stability issues, and immune responses. Some nanomaterials are not biodegradable and their health effects need further study. Ensuring the safety and biocompatibility of nanomaterials in organisms is crucial for their application. Furthermore, there is also a lack of standard criteria and ethical guidelines for assessing nanomedicine delivery systems. For better and faster clinical application, nanomaterials must have excellent biodegradability and biocompatibility. Straightforward and effective nanoparticle designs and standardized quality control measures are essential. Improving the delivery efficiency of nanomedicine is key to achieving precise therapy. Optimizing the size, shape, and surface properties of nanomedicine can enhance their ability to penetrate cell membranes and biofilms.

Currently, there are limitations in the application of nanomaterials, but numerous studies are actively exploring this domain. By thoroughly examining the biocompatibility, metabolism, and excretion of nanomaterials, refining their preparation techniques, improving their stability, and strengthening regulatory oversight, the use of nanotechnology in diagnosing and treating *H. pylori* is expected to accelerate, offering patients additional treatment options.

### 4.6 Limitations

Our study is a bibliometric analysis focusing on *H. pylori*-related nanomaterials research. Nevertheless, there are inevitable limitations. Firstly, the data are limited to the WoSCC and do not include data from other databases like PubMed, Google Scholar, and Cochrane Library. While the WoSCC is comprehensive and credible, using a single database may miss some articles. Additionally, only English-language articles and reviews were included, potentially excluding papers in other languages and early access or proceedings papers, which could lead to biased results. Secondly, variations in the publication year make it difficult to directly compare citations from recently published papers with those from older ones. Supplementary Table S6 lists the top 10 most cited references on *H. pylori* and nanomaterials, but the substantial time interval between publications makes direct citation frequency comparisons biased. Therefore, we have plotted the annual citation of the top 10 most frequently cited references since their publication, as shown in Supplementary Figure S2. However, we are unable to present the annual citation for all articles, which is a limitation of our study. Thirdly, the data may be inconsistent in various aspects. For example, the same institution may use different names at different times.

## 5 Conclusion

This study conducted a comprehensive bibliometric analysis focusing on *H. pylori*-related nanomaterials study. We retrieved and filtered 177 original articles from 2003 to 2023, focusing on the application of nanomaterials in *H. pylori* studies, employing CiteSpace and VOS viewer software for comprehensive visualizations. Notably, China, India, and Egypt are significant contributors to this field. *The International Journal of Biological Macromolecules* and *Biomaterials* are the leading journal, and Yu-hsin Lin is the most important scholar. Our analysis highlights that the development of specific nanoparticles and targeted drug delivery systems remains a burgeoning research area in *H. pylori*-related nanomaterials. In conclusion, our study offers valuable insights into the current state of *H. pylori*-related nanomaterials research and identifies promising avenues for future research.

## Data Availability

Publicly available datasets were analyzed in this study. This data can be found in the Web of Science Core Collection, available here: https://webofscience.clarivate.cn/wos/woscc/summary/c70db6dc-1340-467a-8321-9eb5dffe4006-0154358a7f/relevance/1.
