# Supplementary material for: Research trends of nanomaterials in Helicobacter pylori: a bibliometric analysis from 2003 to 2023
Source: Front Pharmacol. 2025 Mar 28;16:1546395. doi: 10.3389/fphar.2025.1546395 (PMC11986359; doi:10.3389/fphar.2025.1546395)
Supplement: Supplementary file 1 [file DataSheet1.docx]

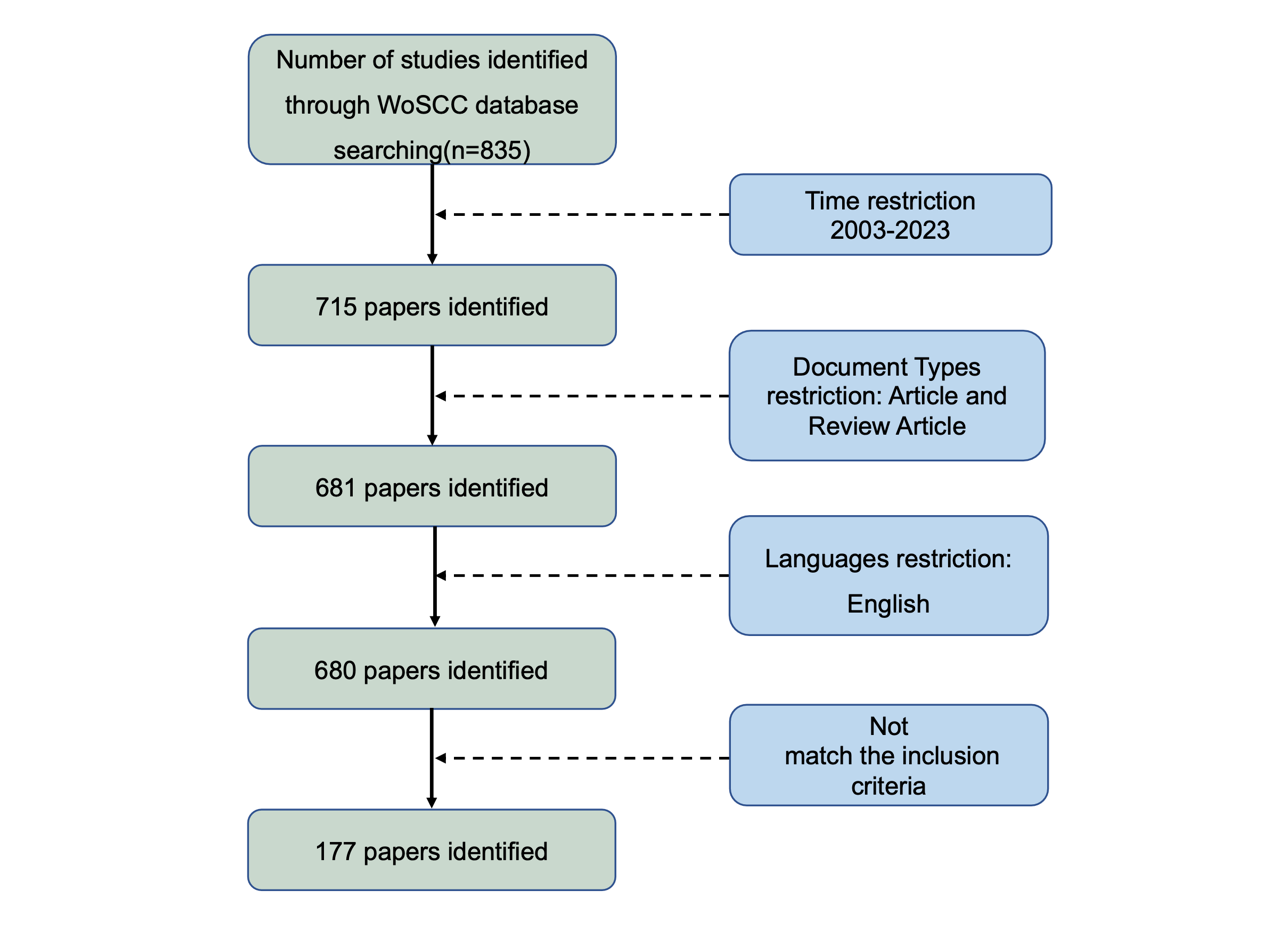


Figure S1 Flow chart of literature screening.

We searched the Web of Science Core Collection (WoSCC) database on October 1, 2024, using the following search formula: (TS = (“*Helicobacter pylori*” OR “*Campylobacter pylori*” OR “*H. pylori*” OR “*Campylobacter pylori subsp. Pylori*” OR “*Campylobacter pyloridis*” OR “*Campylobacter pylori*”)) AND TS = (nano*). A total of 177 original English-language articles on *H. pylori* and nanomaterials are retrieved from 2003 to 2023, encompassing both articles and review articles.


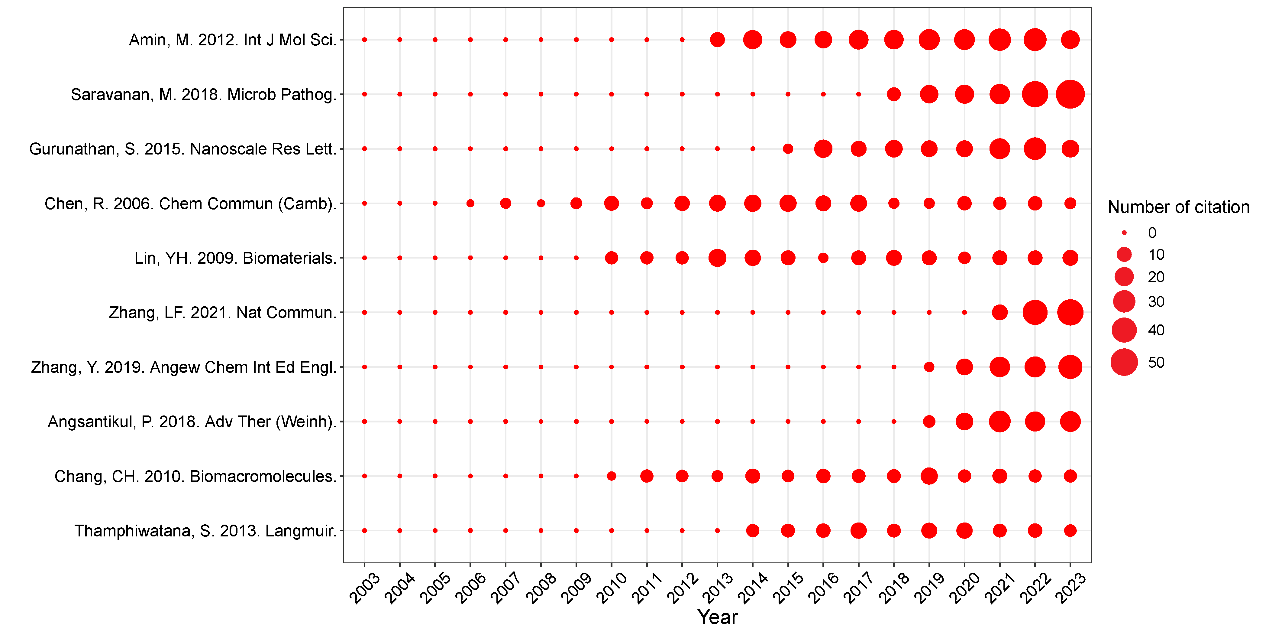


Figure S2 Annual citations of top 10 highly cited references.

The annual number of citations for papers with a high citation score, where the size of the circles represents the citation of the literature. The larger the circles, the higher the citation rates and the greater the influence within the field.

Table S1 Top 10 most productive countries on *H. pylori* and nanomaterial from 2003 to 2023.

| **Rank** | **Country** | **Documents** | **Rank** | **Country** | **Citations** | **Rank** | **Country** | **Total Link Strength** |
| --- | --- | --- | --- | --- | --- | --- | --- | --- |
| 1 | China | 82 | 1 | China | 2840 | 1 | China | 28 |
| 2 | India | 26 | 2 | India | 1225 | 2 | Egypt | 21 |
| 3 | Egypt | 20 | 3 | United States | 687 | 3 | Saudi Arabia | 18 |
| 4 | Iran | 18 | 4 | Malaysia | 509 | 4 | India | 17 |
| 5 | Saudi Arabia | 14 | 5 | Saudi Arabia | 426 | 5 | Pakistan | 14 |
| 6 | Pakistan | 10 | 6 | Pakistan | 410 | 6 | Iran | 9 |
| 7 | United States | 10 | 7 | Iran | 409 | 7 | Malaysia | 8 |
| 8 | Portugal | 8 | 8 | Portugal | 312 | 8 | United Kingdom | 8 |
| 9 | United Kingdom | 7 | 9 | Egypt | 277 | 9 | United States | 5 |
| 10 | Malaysia | 5 | 10 | South Korea | 277 | 10 | Sudan | 5 |

Table S2 Top 10 institutions in terms of number of articles issued and frequency of citations.

| **Rank** | **Institution** | **Documents** | **Original**  **Country** | **Rank** | **Institution** | **Citations** | **Original**  **Country** |
| --- | --- | --- | --- | --- | --- | --- | --- |
| 1 | Ocean Univ China | 9 | China | 1 | Ocean Univ China | 240 | China |
| 2 | Al Azhar Univ | 8 | Egypt | 2 | Qingdao Municipal Hosp | 166 | China |
| 3 | Qingdao Municipal Hosp | 5 | China | 3 | Al Azhar Univ | 159 | Egypt |
| 4 | Cairo Univ | 4 | Egypt | 4 | King Abdulaziz Univ | 95 | Saudi Arabia |
| 5 | Qassim Univ | 4 | Saudi Arabia | 5 | Univ Tehran Med Sci | 57 | Iran |
| 6 | Ain Shams Univ | 3 | Egypt | 6 | Jazan Univ | 52 | Saudi Arabia |
| 7 | Najran Univ | 3 | Saudi Arabia | 7 | Jouf Univ | 52 | Saudi Arabia |
| 8 | Natl Res Ctr | 3 | China | 8 | King Abduallah Int Med Res Ctr | 52 | Saudi Arabia |
| 9 | King Abdulaziz Univ | 2 | Saudi Arabia | 9 | King Saud Bin Abdulaziz Univ Hlth Sci | 52 | Saudi Arabia |
| 10 | Shandong Univ | 2 | China | 10 | Minist Hlth | 52 | Saudi Arabia |

Table S3 Top 10 most productive author and co-cited authors on *H. pylori* and nanomaterial.

| **Rank** | **Author** | **Documents** | **Country** | **Author** | **Co-citations** | **Country** | **Author** | **Total Link Strength** | **Country** |
| --- | --- | --- | --- | --- | --- | --- | --- | --- | --- |
|  |  |  |  |  |  |  |  |  |  |
| 1 | Yu-hsin Lin | 8 | China | Yu-hsin Lin | 84 | China | Yu-hsin Lin | 1672 | China |
| 2 | Muhammad Arif | 7 | China | Peter Malfertheiner | 52 | Germany | Peter Malfertheiner | 1040 | Germany |
| 3 | Zhe Chi | 7 | China | RB Umamaheshwari | 48 | India | Soracha D. Thamphiwatana | 878 | Thailand |
| 4 | Narendra K. Jain | 6 | India | Soracha D. Thamphiwatana | 39 | Thailand | RB Umamaheshwari | 814 | India |
| 5 | Liangfang Zhang | 6 | United States | Francis Megraud | 35 | France | Sahadev D. Ramteke | 735 | India |
| 6 | Weiwei Gao | 5 | United States | David Y. Graham | 34 | United States | David Y. Graham | 719 | United States |
| 7 | Chenguang Liu | 5 | China | Muhammad Amin | 32 | Pakistan | Muhammad Amin | 690 | Pakistan |
| 8 | Claudia Nunes | 5 | Portugal | Daniela Lopes | 28 | Portugal | Catarina L. Seabra | 529 | Portugal |
| 9 | Marygorret Obonyo | 5 | United States | Muhammad Arif | 26 | China | Muhammad Arif | 523 | China |
| 10 | Salette Reis | 5 | Portugal | Zi-wei Jing | 26 | China | Pierre-Louis Bardonnet | 510 | France |

Table S4 the top 10 journals and co-cited-journals on *H. pylori* and nanomaterial from 2003 to 2023.

| **Rank** | **Journal** | **Publications** | **IF**  **(JCR2023)** | **JCR**  **quartile** | **Co-cited-journal** | **Citations** | **IF**  **(JCR2023)** | **JCR**  **quartile** |
| --- | --- | --- | --- | --- | --- | --- | --- | --- |
|  |  |  |  |  |  |  |  |  |
| 1 | *International Journal of Biological Macromolecules* | 7 | 7.7 | Q1 | *Biomaterials* | 225 | 12.8 | Q1 |
| 2 | *Acs Applied Materials & Interfaces* | 5 | 8.3 | Q1 | *J Control Release* | 219 | 10.5 | Q1 |
| 3 | *Journal of Controlled Release* | 5 | 10.5 | Q1 | *Helicobacter* | 205 | 4.3 | Q2 |
| 4 | *Journal of Drug Targeting* | 5 | 4.3 | Q1 | *Int J Pharmaceut* | 164 | 5.3 | Q1 |
| 5 | *Frontiers In Microbiology* | 4 | 4 | Q2 | *World Journal of Gastroenterology* | 162 | 4.3 | Q1 |
| 6 | *International Journal of Nanomedicine* | 4 | 6.6 | Q1 | *Vaccine* | 156 | 4.5 | Q2 |
| 7 | *International Journal of Pharmaceutics* | 4 | 5.3 | Q1 | *Adv Drug Deliver Rev* | 152 | 15.2 | Q1 |
| 8 | *Molecular Pharmaceutics* | 4 | 4.5 | Q2 | *Gastroenterology* | 152 | 25.7 | Q1 |
| 9 | *Pharmaceutics* | 4 | 4.9 | Q1 | *Gut* | 138 | 23.0 | Q1 |
| 10 | *Biomaterials* | 3 | 12.8 | Q1 | *Infect Immun* | 132 | 2.9 | Q2 |

Table S5 Top 20 keywords on *H. pylori* and nanomaterial from 2003 to 2023.

| **Rank** | **Keyword** | **Occurrences** | **Total link strength** | **Rank** | **Keyword** | **Occurrences** | **Total link strength** |
| --- | --- | --- | --- | --- | --- | --- | --- |
|  |  |  |  |  |  |  |  |
| 1 | *H. pylori* | 129 | 752 | 11 | silver nanoparticles | 20 | 125 |
| 2 | nanoparticle | 91 | 579 | 12 | treatment | 16 | 120 |
| 3 | drug-delivery | 59 | 421 | 13 | clarithromycin | 15 | 110 |
| 4 | infection | 54 | 335 | 14 | triple therapy | 15 | 121 |
| 5 | in-vitro | 44 | 305 | 15 | acid | 13 | 86 |
| 6 | chitosan | 40 | 272 | 16 | adhesion | 13 | 105 |
| 7 | antibiotic-resistance | 36 | 209 | 17 | biofilm | 11 | 77 |
| 8 | antibacterial | 33 | 189 | 18 | system | 11 | 78 |
| 9 | eradication | 27 | 200 | 19 | toxicity | 10 | 63 |
| 10 | Amoxicillin | 25 | 190 | 20 | cancer | 9 | 60 |

Table S6 Top 10 high-cited references regarding *H. pylori* and nanomaterial.

| **Rank** | **Authors** | **Article Title** | **Source Title** | **Citations** | **Year** | **Document Type** | **DOI** |
| --- | --- | --- | --- | --- | --- | --- | --- |
| 1 | Amin, M et al. | Green Synthesis of Silver Nanoparticles through Reduction with Solanum xanthocarpum L. Berry Extract: Characterization, Antimicrobial and Urease Inhibitory Activities against *Helicobacter pylori* | *Int J Mol Sci* | 235 | 2012 | Article | 10.3390/ijms13089923 |
| 2 | Saravanan, M et al. | Green synthesis of anisotropic zinc oxide nanoparticles with antibacterial and cytofriendly properties | *Microb Pathog* | 172 | 2018 | Article | 10.1016/j.micpath.2017.12.039 |
| 3 | Gurunathan, S et al. | Multidimensional effects of biologically synthesized silver nanoparticles in *Helicobacter pylori*, Helicobacter felis, and human lung (L132) and lung carcinoma A549 cells | *Nanoscale Res Lett* | 149 | 2015 | Article | 10.1186/s11671-015-0747-0 |
| 4 | Chen, R et al. | Fabrication of bismuth subcarbonate nanotube arrays from bismuth citrate | *Chem Commun (Camb)* | 145 | 2006 | Article | 10.1039/b601764a |
| 5 | Lin, YH et al. | Development of pH-responsive chitosan/heparin nanoparticles for stomach-specific anti-*Helicobacter pylori* therapy | *Biomaterials* | 134 | 2009 | Article | 10.1016/j.biomaterials.2009.02.036 |
| 6 | Chang, CH et al. | Nanoparticles Incorporated in pH-Sensitive Hydrogels as Amoxicillin Delivery for Eradication of *Helicobacter pylori* | *Biomacromolecules* | 107 | 2010 | Article | 10.1021/bm900985h |
| 7 | Zhang, Y et al. | Inhibition of Pathogen Adhesion by Bacterial Outer Membrane-Coated Nanoparticles | *Angew Chem Int Ed Engl* | 103 | 2019 | Article | 10.1002/anie.201906280 |
| 8 | Angsantikul, P et al. | Coating Nanoparticles with Gastric Epithelial Cell Membrane for Targeted Antibiotic Delivery against *Helicobacter pylori* Infection | *Adv Ther (Weinh)* | 98 | 2018 | Article | 10.1002/adtp.201800016 |
| 9 | Zhang, LF et al. | In vivo activation of pH-responsive oxidase-like graphitic nanozymes for selective killing of *Helicobacter pylori* | *Nat Commun* | 97 | 2021 | Article | 10.1038/s41467-021-22286-x |
| 10 | Thamphiwatana, S et al. | Nanoparticle-Stabilized Liposomes for pH-Responsive Gastric Drug Delivery | *Langmuir* | 93 | 2013 | Article | 10.1021/la402695c |
